# SOC estimation for a lithium-ion pouch cell using machine learning under different load profiles

**DOI:** 10.1038/s41598-025-02709-1

**Published:** 2025-05-24

**Authors:** J. Harinarayanan, P. Balamurugan

**Affiliations:** School of Electrical Engineering, Vellore Institute of Technology, Chennai, 600127 India

**Keywords:** Li-ion pouch cell, State of charge, Load profile, Coulomb counting, Machine learning, Electrical and electronic engineering, Batteries

## Abstract

Estimating the state of charge of lithium-ion battery systems is important for efficient battery management systems. This work conducts a thorough evaluation of multiple SOC estimate methods, including both classic approaches Coulomb Counting and extended Kalman filter and machine learning techniques under different load profile on lithium-ion pouch cell. The assessment included a variety of experimental data collected from entire cycles, shallow cycles, and dynamic operations utilizing the Worldwide Harmonized Light Vehicles Test Procedure and Hybrid Pulse Power Characterization tests done from 100% to 10% SOC. While traditional approaches performed well under ordinary settings, they had severe limits during shallow cycling. In contrast, machine learning technologies, notably the random forest method, performed better across all testing conditions. The random forest approach showed outstanding accuracy while minimizing error metrics (RMSE: 0.0229, MSE: 0.0005, MAE: 0.0139) and effectively handled typical issues such as SOC drift and ageing effects. These findings validate random forest as a dependable and robust approach for real-time SOC estimation in battery management systems.

## Introduction

Accurate State of Charge (SOC) assessment in lithium-ion batteries is essential for the maximum performance and durability of battery-powered devices, especially in electric cars and energy storage systems. SOC represents the remaining charge in the battery, which must be preserved to enhance battery performance, manage charging and discharging processes, and guarantee safety. Recent advancements have offered many SOC estimation algorithms; nonetheless, Coulomb Counting and the Extended Kalman Filter (EKF) remain the most conventional methods, extensively used in battery management systems (BMS). Coulomb Counting is a direct approach that sums current over time to ascertain the State of Charge; nevertheless, this methodology is susceptible to significant mistakes owing to factors such as ageing, temperature fluctuations, and measurement noise^1^. In contrast, the EKF is a more advanced method that overcomes the shortcomings of Coulomb Counting by using a recursive state estimate strategy that combines model-based predictions with empirical data. While both traditional methods provide adequate accuracy, they often exhibit suboptimal performance, especially in shallow cycles or dynamic operational situations^2^. Using the Unscented Kalman Bucy Filter (UKBF) in^3^ the authors provide a SOC estimate technique coupled with a 2RC Thevenin battery model. Compared to conventional techniques like EKF and UKF, the UKBF effectively manages noise and nonlinearities, therefore attaining better SOC accuracy. The system is verified in MATLAB Simulink showing better SOC estimating performance. By handling complicated correlations in battery activity, which conventional methods fail to handle, ML algorithms have recently shown potential in enhancing State of Charge estimates. Random Forest (RF), a prevalent ensemble learning method, has shown significant effectiveness in several predictive applications, including State of Charge estimate^4^. Also the Machine learning-based SOC estimate outperforms more conventional approaches on real-world driving data, demonstrating its robustness and reliability in real-world settings. Machine learning offers more precise and trustworthy SOC estimate than conventional approaches, which typically include inaccuracies when evaluated against real-world driving circumstances^5^ By using an ensemble of decision trees, Random Forest may discern intricate patterns within the data. This alleviates problems often associated with traditional methods, like SOC drift and ageing effects. Safe and efficient lithium-ion battery operation in electric cars requires precise State of Charge measurement^5,6^.

## Literature review

Coulomb counting, a widely used method, determines state of charge by integrating current over time. The method’s vulnerability to cumulative mistakes is a major drawback, especially in contexts that are constantly changing. To solve these problems, many methods have been suggested. Traditional approaches used calculations based on currents, which are both computationally intensive and susceptible to measurement errors^7^. Coulomb counting, while straightforward, has accuracy issues in dynamic scenarios, leading to the creation of model- based methods like the Kalman filter method^8^. Electric vehicle batteries by limiting overcharging and over discharge while also providing critical cell balance. Accurate estimate of the state of charge is censorious for improving battery performance and lifespan. To accomplish this, a variety of SOC estimating approaches are used, including direct measurement, bookkeeping, adaptive, and hybridstrategies, each with its own set of benefits and drawbacks^9^. SOC by machine learning methods has the system dynamics to improve State of Charge estimations; however, data-driven algorithms such as Support Vector Machines (SVM) regression, linear regression, and random forests offer flexibility and effectiveness in various contexts, albeit requiring extensive datasets for training^10^. The use of deep neural networks (DNNs) and transfer learning improves the model’s generalizability to various battery types, temperatures, and aging scenarios for estimating SOC. To simplify retraining, you may use pre-trained models like as convolutional neural networks (CNNs), long short-term memories (LSTMs), and transformers to capture complicated battery dynamics. As a result, SOC forecast accuracy is enhanced, making it more suitable for practical use^11^. LSTM and CNN networks are constructed in a data-driven capacity estimate approach. While the CNN finds local characteristics to improve capacity prediction, the LSTM records temporal dependencies. This variant greatly lowers RMSE, therefore enhancing the dependability of battery condition monitoring. Presenting in a machine learning-based problem diagnostic system for electric vehicle lithium-ion batteries is reaching great accuracy, the system uses SVM, RF, and KNN algorithms with PCA for fault classification. It is a strong answer for battery problems as it identifies and categorizes battery failures in real time^12^.

Recent research have emphasized the usefulness of voltage-based approaches to improve estimation accuracy and efficiency. The state of charge also can be estimated by advances previous voltage-based methods by introducing a self-calibrating algorithm that utilizes a fundamental equivalent circuit model for correct SOC estimation, demonstrating improved resilience under ageing circumstances and decreased computational complexity in comparison to current techniques^13^. Furthermore, typical ampere-hour integration methods for state of charge assessment exhibit a decline in accuracy over time when operating in the presence of Gaussian noise. Thevenin equivalent circuit models, together with EKF, have been used to enhance estimate robustness and accuracy by reducing the impact of noise^14–16^. Machine learning techniques have shown significant promise for State of Charge estimation. Research comparing traditional techniques, such as Coulomb counting and Open Circuit Voltage (OCV) methods, with machine learning algorithms demonstrates that the latter provides superior accuracy, with average errors under 1%, making machine learning a compelling option for battery management systems^17^. This work introduces a dynamic SOC estimation approach that aligns battery energy output with fluctuating load power profiles, therefore improving SOC accuracy across various discharge conditions^18^.

The increasing predominance of electric vehicles presents significant problems to the electrical grid, and precise estimation of state of charge is crucial for mitigating these concerns. Hybrid approaches including corrective strategies, such as enhanced Open Circuit Voltage resets and iterative calculations of Direct Current Internal Resistance (DCIR), have been proposed to mitigate computational complexity and inaccuracies in State of Charge. These strategies enhance the effectiveness of Coulomb counting in diverse operating environments, improving state-of-charge assessments in real- time battery management systems^19^.

Innovations in battery technology are urgently needed to ensure a sustainable transportation system can keep up with the rapid expansion of electric automobiles. Ensuring the battery’s effectiveness, drivability, and protection relies on precisely estimating its temperature and charge level. This work provides an AI-based methodology for SOC and temperature estimates that uses a variety of regression models. The results provide light on battery temperature behavior, helping to build a more effective battery management system^20^. The Fixed-Interval Discrete Extended Kalman Filter (FDEKF) approach uses Hybrid Pulse Power Characterization (HPPC) for parameter identification and validation, ensuring a reliable State of Charge estimation across various operating conditions. This method has shown improved predictive accuracy for lithium-ion batteries, addressing the intricacies of load dynamics and strengthening status of charge monitoring in real-world electric vehicle applications^21^.

The integration of electric vehicles into the grid requires effective management of charging infrastructure. The evaluation of State of Charge, when combined with real-time data and Geographic Information Systems (GIS), might improve the identification of charging station locations and predict high- demand areas in urban environments. This approach improves the effectiveness of electric vehicle infrastructure and supports advanced energy management systems^22^.

A novel state of charge estimate method that integrates a self-calibrating voltage-based approach with a basic equivalent circuit model, offering a dependable solution for accurate SOC and state of health (SOH) assessment across various operating conditions. This method demonstrates significant improvements in estimation accuracy and computational efficiency via thorough testing, establishing it as a feasible alternative to traditional SOC estimation techniques^23^. We provide a thorough investigation of SOC estimate in this work by comparing and contrasting a number of approaches, including both conventional methods like Extended Kalman Filter and Coulomb Counting and ML models. Adaptability and robustness in real-world settings are limited since prior research mainly concentrate on SOC estimates using either classical techniques or ML approaches under a single load profile, according to the literature review. Under addition, there are a number of drawbacks to CC and EKF, which make them imperfect under dynamic operating situations, sensitive to noise, and frequently employed because of their simplicity and real-time application. The variety of the training data has a major impact on the efficacy of ML algorithms, which capture complicated battery behaviors and display superior predicting accuracy.

Our goal is to address the shortcomings of earlier research by estimating SOC under a variety of real-world operating scenarios utilizing four different load profiles: full load, partial load, dynamic load, and HPPC. To mimic heavy use, the full load profile continuously discharges the battery at a high power. To reflect real-world operating conditions, the partial load profile records moderate and intermittent loading cycles. While the dynamic load profile mimics actual driving situations with fluctuating power and current demands, the HPPC profile estimates internal resistance and captures the transient response characteristics of the battery by applying brief, high-power pulses. Using these varied load profiles, we compare the accuracy and robustness of conventional approaches with those of ML models. The experimental findings show that ML-based approaches, especially Random Forest, are more trustworthy for SOC prediction under dynamic situations than classical methods because to their higher accuracy and reduced estimate errors. When it comes to capturing complicated battery dynamics, Random Forest ML method regularly outperforms the others, with the lowest error rates. A comparative review of multiple SOC calculation methods used to various lithium-based battery cell types under varied load profiles is given in the Table 1. It encompasses both contemporary machine learning techniques as well as traditional techniques like observer-based models and Kalman filters. Metrics like MAE and RMSE are used to quantify performance, and a number of approaches achieve high accuracy (less than 1% inaccuracy). The suggested approach performs competitively under dynamic, HPPC, full, and partial load scenarios.

An innovative feature that improves the SOC estimate models’ capacity for generalization is the work’s multi-profile assessment approach. This study shows that the model’s dependability and resilience are much enhanced when trained and tested over several varied profiles, in contrast to earlier research that only considers one load profile. The findings demonstrate that the ML models, especially Random Forest, can reliably forecast SOC in all real-world scenarios after being trained on these different profiles. This makes the suggested approach ideal for use in BMS and EVs. Ultimately, error metrics are evaluated. Error measures were assessed by different methods, including Root Mean Square Error (RMSE), Mean Square Error (MSE) and Mean Absolute Error (MAE).

**Table 1:** Comparison of the literature review.

References	Algorithm used	Type of cell used	Load profile used	Performance metrics
^24^	Effective medium model (EMM)	10 Ah NMC pouch cell	Laser Doppler vibrometry at 0% and 100% SOC for more than 80 cycles.	2.7% for 100% SOC 13.6% for 0% SOC
^25^	Dissipativity observer approach	A123 20Ah pouch cell	HPPC test	Not mentioned
^26^	Deterministic ensemble Kalman-Bucy filter (DEnKBF) and Ensemble Kalman-Bucy filter (EnKBF) and	NMC battery	HPPC test	MAE of 6.9 × 10^−4^, 2.23 × 10^−3^ and RMSE of 1.2 × 10^−3^, 2.41 × 10^−3^
^27^	Modified adaptive extended Kalman filter (MAEKF)	TurnigyGraphene5Ah65C cell	LA92, US06 and mixed drive cycle	RMSE and MAE for LA92: 0.891 & 1.390 for 0 °C , 1.254 & 1.489 for 25 °C , 1.012 & 1.293 for 40 °C for US06: 2.997 & 4.114 for 0 °C, 2.568 & 3.405 for 25 °C , 1.379 & 3.217 for 40 °C
^28^	GAMIUKF (Genetic Algorithm-Multiinnovation Unscented Kalman Filter) algorithm	Lead–carbon batteries	HPPC, UDDS discharge test	Average error of 2%
^29^	Support Vector Machine, Gaussian Process Regression (GPR) Artificial Neural networks (ANN),	8 Ah LiPF_6_ Pouch cell	Performance under temperature and load stress	< 4%
^30^	Feed-Forward Artificial Neural Network	NMCLMO blended 15Ah Pouch cell	Full load and partial load profile	RMSE of 1.17% for discharge and1.81% over charge profile
^31^	Long short-term memory (LSTM) network	NCM811 14Ah cell	Under constant current profile (CC), urban dynamometer driving schedule (UDDS) and Dynamic stress test (DST)	MAE 0.34% and RMSE 0.45%
^32^	Sliding mode observer with adaptive switching gain	64 Ah LIB pouch cell	Pulse current and UDDS	< 1% for fresh cell and < 3% for aged cell
^33^	A modified Coulomb counting method based on regional temperature features	10 Ah pouch cell	Charging and discharging at different temperature	MAE of 1–2.5%
^34^	Charging characterization	19Ah lithium-sulfur pouch cell	Charging, discharging and HPPC	< 3%
^35^	Spatial restoration algorithm and dual Kalman filter (DKF).	5 Ah	WTLC, NEDC	1.65 for WTLC 2.12 for NEDC
Proposed	Coulomb counting, EKF, Linear regression, Support vector machine, Random forest	26Ah lithium ion pouch cell	Full load, partial load, dynamic load and HPPC	RMSE, MSE, MAE as 0.0229, 0.0005, 0.0139

## Estimation methods

**Fig. 1:**
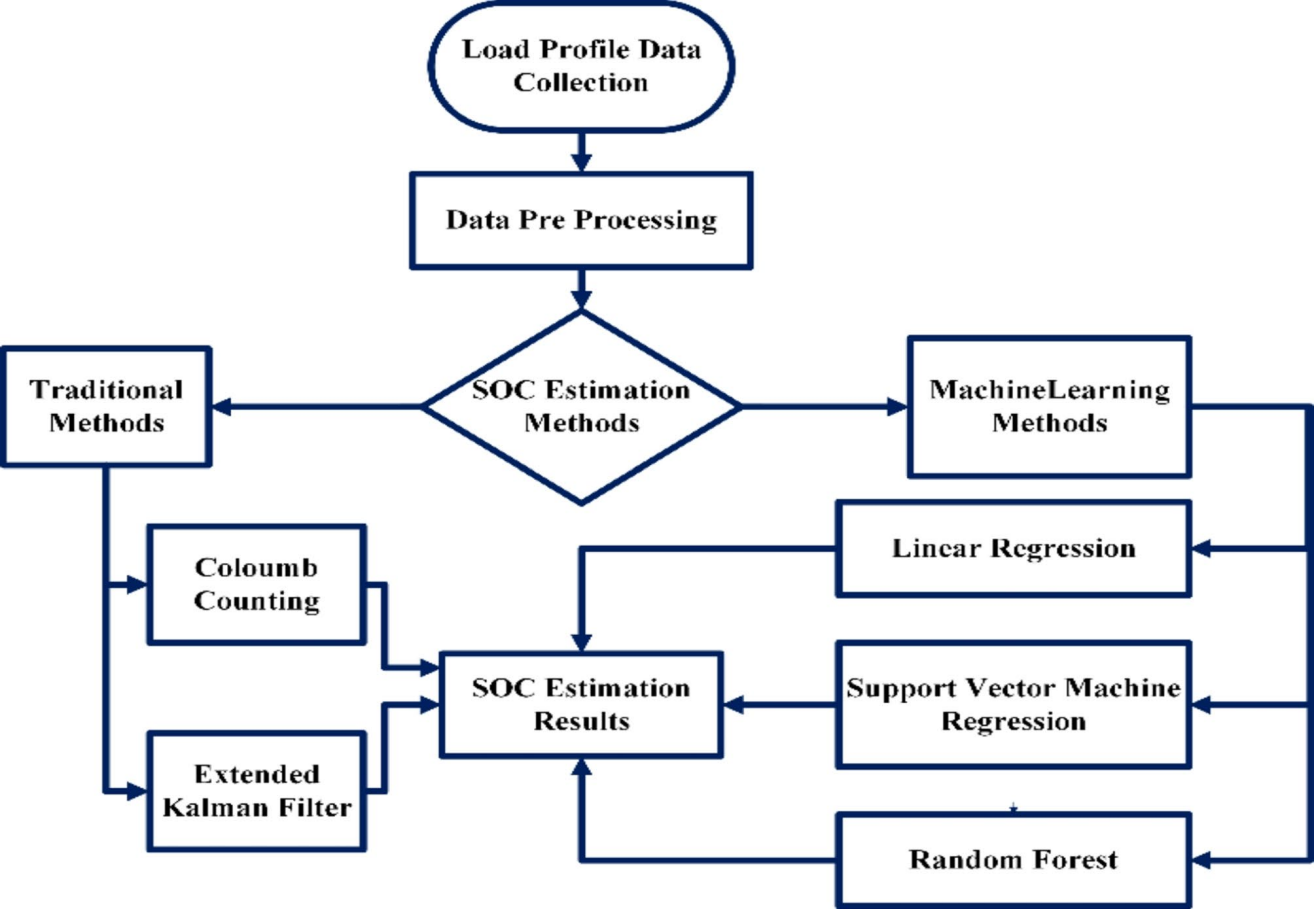
Work flow process.

The Soc estimated by both the traditional and the machine learning methods. Figure 1 depicts the SOC estimation process, which begins with the gathering of load profile data and continues with data preparation to improve the input for analysis. Traditional approaches and machine learning models are used for estimate, with each providing varying accuracy and complexity trade- offs. Finally, the estimated SOC values are analyzed to assess performance, contrasting the dependability of conventional methods with the increased accuracy of machine learning techniques.

A. **Coulomb counting method**
  Coulomb counting estimates SOCs by measuring and integrating current over time. Battery cells must retain their State of Charge throughout charge, discharge, and storage to operate safely. Instead of measuring SOC directly, various measurements and factors are used to approximate it. This causes estimated SOC inaccuracies, preventing full cell utilization.Coulomb counting gives a relative and not absolute SOC change. Current measurements across time steps determine the quantity of Ah that left or entered the battery. Equation for Coulomb counting:
  1 $$\text{SOC}\left(\text{t}\right)=\text{SOC}\left(\text{t}-1\right)+\frac{1}{{\text{Q}}_{\text{n}}}\int\limits_{\text{t}-1}^{\text{t}}\text{I}\left(\text{t}\right)\text{dt}$$
  where SOC(t) is the SOC assessed at time ‘t’. SOC(t-1)is the previous SOC. I(t) is the current of charging (negative) or discharging (positive). Q_n_ represents the cell’s nominal capacity. dt is the time for each step.
  To obtain an absolute SOC, the initial SOC level for a particular cell must be known. A practical way to achieve this is by charging to the maximum voltage to reach the maximum cell SOC, allowing a reset point to be established. The Coulomb Counting technique may exhibit inaccuracies due to initial State of Charge assumptions, current measurement precision, current integration errors, battery capacity uncertainties, and timing discrepancies.
B. **Extended Kalman filter method**
  The Extended Kalman Filter is a nonlinear state estimation method that enhances the classic Kalman Filter (KF) to accommodate nonlinear system dynamics. It is extensively used to estimating the charge remaining in the battery through proper battery management systems due to its efficacy in addressing the nonlinear properties of battery models. The EKF is an effective technique for State of Charge estimate, using battery models and real-time voltage measurements to enhance precision^36^. By persistently refining State of Charge forecasts with actual voltage measurements, it surpasses more rudimentary techniques such as Coulomb Counting alone. In the case of SOC estimation, the state vector x (a) includes SOC:
  2 $$\text{x}(\text{a})=\left[\begin{array}{l}\text{SOC}\left(\text{a}\right)\\ \:\text{Other}\:\text{Parameters}\end{array}\right]$$
  The state equation f (x, u) models SOC dynamics using Coulomb Counting:
  3 $$\:\text{S}\text{O}\text{C}\left(\text{a}+1\right)=\text{S}\text{O}\text{C}\left(\text{a}\right)-\:\frac{{\upeta\:} \cdot {\Delta\:}\text{t}}{\text{C}}\text{I}\left(\text{a}\right)$$
  where η is the Columbic efficiency. C is the battery capacity. I(a) is the current (positive for discharge, negative for charge).
  The measurement equation h(x, u) relates SOC to terminal voltage using an equivalent circuit model (ECM):
  4 $${\text{V}}({\text{a}}) = {\text{h}}({\text{SOC}}({\text{a}}), \quad {\text{I}}({\text{a}})) + {\text{v}}({\text{a}})$$
  where V(a) is the measured voltage. I(a) is the current flowing through the battery. v(a) is measurement noise or errors in the voltage measurement.
C. **Machine learning methods**
  The use of supervised learning specifically in machine learning has enhanced the State of Charge estimation in lithium-ion batteries. This is because ML can handle the non-linear behavior of the battery and provide accurate predictions under a variety of operating scenarios^37,38^. SOC estimation is often employed in this paper as follows: Linear Regression and Support Vector Machine Regression (SVR) are both powerful techniques for predictive modeling. SVR utilizes kernel functions such as Radial Basis Function (RBF), polynomial, and linear to convert input data into a higher-dimensional space, facilitating more accurate predictions and the identification of complex patterns. The model’s performance relies on setting hyper parameters such as regularization (C), error tolerance (ε), and kernel coefficient (γ), which requires feature scaling via standardization or normalization. Cross-validation, usually k-fold, selects the best hyper parameters to prevent overfitting and guarantee resilience. Random Forest Regression, which uses numerous decision trees, is another effective SOC estimating approach. Its characteristics, significance ranking, strong resilience to outliers and noise, and the ability to handle numerical and categorical characteristics make this technique suited for complicated datasets. The regression techniques to forecast the SOC of batteries employed in Electric Vehicles (EVs). A Current Integrating method is used for SOC assessment, and the model is constructed and evaluated utilizing many drive cycles (FTP-75, HWFET, US06, and UDDS) in Matlab/Simulink to provide datasets for model training. The efficacy of various regression models is evaluated using Root Mean Square Error (RMSE), with neural networks demonstrating superior performance. The research indicates that the neural network model, including 15 neurons, yields the minimal RMSE, surpassing other models such as ensemble bagged trees, Support Vector Machines, and binary tree regressions. The presented model exhibits precise State of Charge prediction with minimum sensor prerequisites^39^.

1. **Linear regression method**
  A supervised machine learning technique called linear regression finds the best-fitting linear function for mapping data points using labeled datasets. By establishing a linear connection between the dependent variable and one or more independent variables, predictive modelling for fresh datasets is enabled. This method applies a linear equation to observed data, allowing for the estimation of continuous output variables using provided input characteristics. It is a fundamental and extensively used supervised learning technique for determining SOC. It presupposes a linear correlation between input characteristics (such as temperature, voltage, current, etc.,) and the state of charge^40^. The linear regression formula may be expressed as:
  5 $$y = {\alpha _0} + {\alpha _1}{x_1} + {\alpha _2}{x_2} + \cdots + {\alpha _n}{x_n} + \varepsilon$$
  where y is the predicted SOC. α_0_ is the intercept. α_1_, α_2_,…α_n_ are the regression coefficients (weights). x_1_, x_2_,…x_n_ are the input features (e.g., voltage, current, temperature). ε is the error term.
  Linear regression determines the best-fit line by minimizing errors. It computes the total of the squared differences in expected and actual SOC values. This reduces the total discrepancy, resulting in optimum estimate.
2. **Support vector machine regression (SVR)**
  SVR is a sophisticated method that identifies the hyper plane that optimally fits the data while maximizing the margin of separation. In SOC estimation, Support Vector Regression endeavors to map input features into a higher-dimensional space through the application of a kernel function, then employing linear regression inside that space^41^. The SVR objective is to minimize the following loss function.
  6 $${\frac{1}{2}||{\omega}||}^{2}+\text{a}\sum_{\text{i}=1}^{\text{n}}{{\epsilon}}_{\text{i}}^{2}$$
  where ω is the weight vector. a is the regularization parameter serves to regulate the balance between the magnitude of the margin and the training error. ε_i_ are the slack variables, which represent the deviation from the epsilon-insensitive tube.
  The decision function for predicting SOC is given by:
  7 $$\text{f}\left(\text{x}\right)=\sum_{\text{i}=1}^{\text{n}}{{\alpha}}_{\text{i}}\text{K}({\text{x}}_{\text{i}},\text{x})+\text{k}$$
  where α_i_ are the Lagrange multipliers. K(xi, x) is the kernel function, such as RBF, polynomial, or linear. k is the bias term.
3. **Random Forest regression method**
  Random Forest Regression is an ensemble learning method that integrates several decision trees to provide more reliable and precise predictions. It is very proficient in managing noisy data, outliers, and intricate interactions between the input characteristics and the target SOC^42^. In recent years, machine learning approaches, notably Random Forest, have gained popularity for SOC prediction owing to their capacity to handle nonlinear battery behavior. However, the accuracy of RFs is largelydependent on the right selection of hyper parameters, such as the number of trees and their leaves. To improve RF performance, optimization tools like as the Gravitational Search Algorithm (GSA) have been used to precisely fine-tune these parameters. RFs optimized using GSA outperform traditional RFs and neural network-based models such as Radial Basis Function Neural Networks (RBFNN) in terms of accuracy and resilience across temperature ranges. This development greatly minimizes estimate errors, making it a potential method for improving SOC prediction in EV applications^43^.
  The Random Forest methodology may be succinctly stated as follows:
  Construct many decision trees using random portions of the training dataset.
  Utilize a collection of input attributes for each decision tree to segment the data and forecast the SOC.
  The ultimate SOC prediction is derived by averaging the forecasts of all individual decision trees.
  Mathematically, the Random Forest prediction is:
  8 $${\text{Y}}_{\text{R}\text{F}}=\frac{1}{\text{N}}\sum_{\text{t}=1}^{\text{N}}{\text{f}}_{\text{t}}\left(\text{k}\right)$$
  where, Y_RF_ is the random forest output prediction. N represents the total number of individual decision trees that make up the random forest model. f_t_(k) represents the prediction output generated by the t-th tree in the forest, where t ranges from 1 to N. k refers to the input feature vector.

## Experimental data and simulation

The experimental setup conducted for a lithium-ion pouch cell used in the load vehicles and passenger vehicles which has a nominal voltage of 3.6 V and a nominal capacity of 26Ah A123 pouch cell employing NMC lithium-ion chemistry has an NMC cathode and a graphite anode. The NMC cathode provides elevated energy density and longevity, whereas the graphite anode guarantees consistent lithium-ion intercalation^44^. This amalgamation delivers a harmonious performance for electric vehicles and energy storage systems. The cell features a graphite anode and a cathode composed of a blend of lithium-ion combination batteries such as LMO and NMC batteries. This material combination offers an optimal mix of high energy density, stability, and cycle longevity, making it suitable for the estimation of SOC^45^. All the simulation done using MATLAB in which the data are collected from the test conducted the structured laboratory. The battery is tested in various operating scenarios to determine its performance and behavior. The State of Charge estimate procedure relies on a dataset that includes a wide range of operating situations and battery behaviors. To ensure this, 262,469 data points were gathered from different load profiles. This dataset is comprehensive, allowing for enhanced model accuracy.

**Fig. 2:**
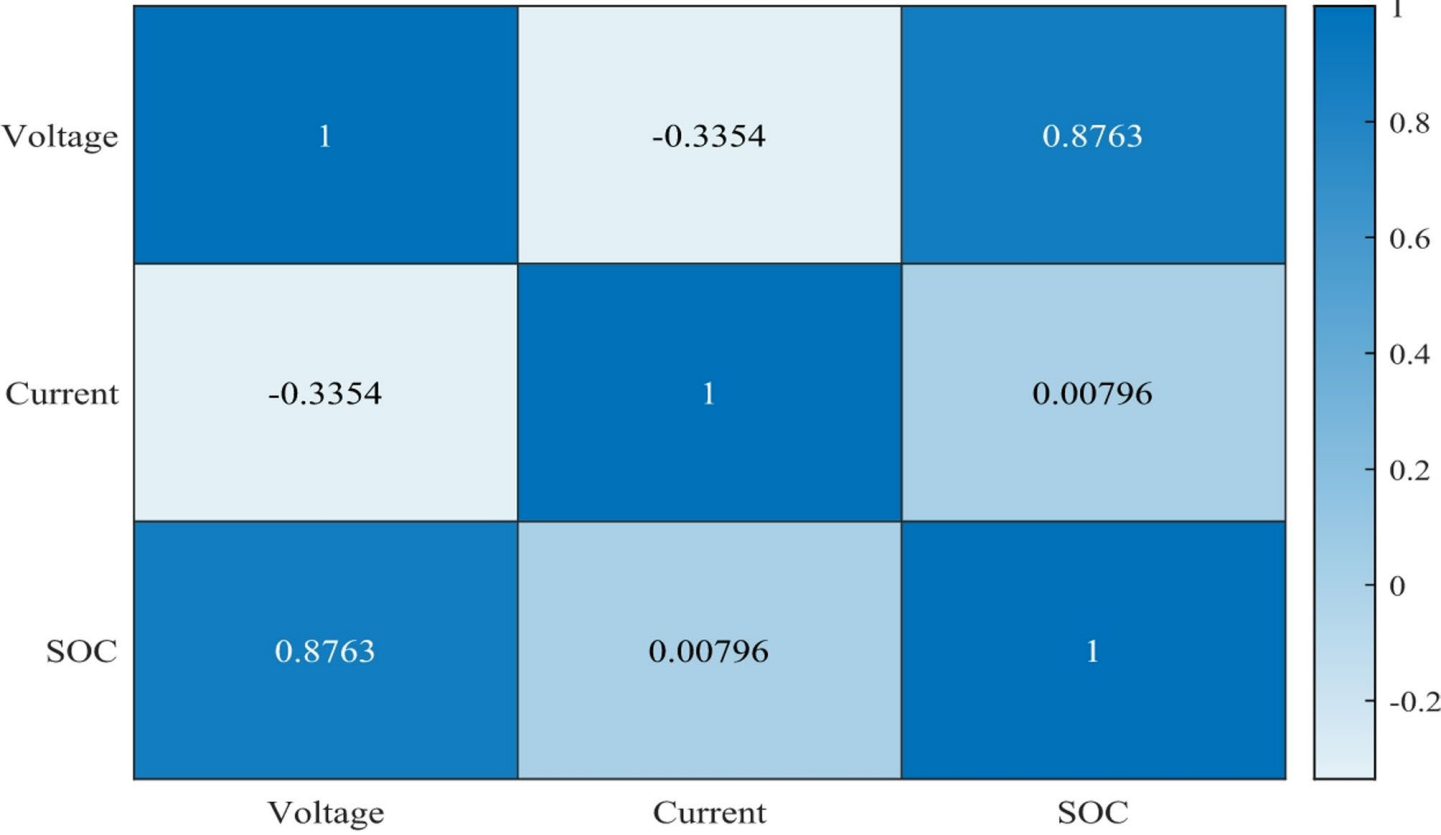
Data feature correlation heat map.

Figure 2 shows the significant correlations are shown by the correlation analysis of the battery parameters. Voltage and State of Charge have the greatest correlation at 0.8763, meaning that voltage is a reliable predictor of battery charge level. A small negative correlation (−0.3354) exists between voltage and current, which reflects the fact that higher currents may lead to lower voltages. It is confirmed that changes in SOC are dependent on current over time rather than immediate values, since there is essentially no connection between current and SOC (0.00796). Based on these results, it seems that current data is better for understanding the behavior of dynamic batteries, whereas voltage measurements are more useful for determining SOC in battery management systems.

The model parameters are estimated using the incremental open-circuit voltage profile, the Trust-Region Reflective method, and the Non-Linear Least Squares approach, which produces an accurate representation of battery dynamics across a range of operating conditions. The predicted parameters are experimentally confirmed using independent dynamic current profiles, such as the Dynamic Stress Test (DST), Federal Urban Driving Schedule (FUDS), Beijing Dynamic Stress Test (BJDST), and the US06 driving cycle, at three temperature levels^46^.

A total of 262,469 data points were obtained during the data gathering method, which included subjecting a lithium-ion battery to four different load profiles utilizing a battery testing equipment. The Full Load Profile (1–53,068 data points) was the first profile that completely charged and drained the battery in one hour by continuously cycling the charge and discharge current at a 1 C rate. Results showed that the battery performed better under continuous load circumstances and that its efficiency and capacity fading were better understood. Instead of doing complete cycles, the battery was charged and discharged at different state-of-charge levels in the second profile, called the Partial Load Profile (53,069–1,39,058 data points). Instead of cycling between 0% and 100% as the full load profile does, this test mimics real-world use by having the battery run at mid-range state of charge (SOC). It was useful for learning how partial charging and discharging affect the lifespan and performance of batteries. To simulate real-world scenarios like those in EVs and consumer electronics, the Dynamic Load Profile (1,39,059–217,000 data points) induced unexpected changes in load. In order to study thermal reactions, voltage recovery, and fluctuations in internal resistance under dynamic settings, researchers used this profile, which includes fast changes in power demand, frequent charging and draining events, and changing current loads. The Hybrid Pulse Power Characterization (HPPC) Test, which requires data from 217,001 to 262,469 points, was used to evaluate the battery’s resistance, power capabilities, and deterioration under pulse settings. The test started with the battery at 100% state of charge and proceeded to deplete it down to 10% in 10% increments. The internal resistance and transient behavior of the cell were assessed at each phase by applying brief pulses of charge and discharge. Because of the vital need of knowing how a battery reacts to pulse loads, the HPPC test finds extensive use in electric and hybrid vehicles.

Here three machine learning methods such as Linear Regression, Support Vector Machine, and Random Forest were trained on 262,469 data points gathered from various load profiles to forecast battery SOC. The data was preprocessed, cleaned missing values, scaled numerical characteristics like voltage and current, and aligned time-series data using resampling methods before training. The dataset was then divided into 80% training, 10% validation, and 10% test sets for estimation. Along with derivative features like dV/dt and dSOC/dt, feature engineering included essential factors such voltage, current, SOC thereby capturing variations across time. Using Ordinary Least Squares (OLS), Linear Regression was trained from a baseline model; while its performance was constrained by the assumption of a linear connection. Nonlinear relationships were captured using Support Vector Machine, and kernel selection—linear and RBF—is mostly responsible for raising accuracy. Regularizing parameter (C) and gamma (γ) in the RBF kernel were optimized using grid search with five-fold cross-valuation (CV). Using many decision trees, Random Forest (RF) an ensemble model was trained with average producing final predictions. Randomized Search CV was used to hyper parameter tune number of trees (n_estimators), tree depth (max_depth), and feature selection (max_features) thereby improving performance while controlling computational cost. A thorough dataset covering the lithium-ion cell’s behavior across numerous operating circumstances was generated using this structured testing technique. Improving battery management systems and extending battery longevity are both aided by these data points, which provide useful insights for modeling, performance assessment, and degradation study of batteries.

## Different load profiles

The four different load profiles—full load, partial load, dynamic load, and HPPC—are used to estimate the state of charge of a pouch cell in this study. To train the ML model, we need a wide and complete dataset, and each profile represents a different operational state. In the full load profile, high power is discharged continuously to reflect heavy use, while in the partial load profile, moderate loading is simulated by intermittent cycles. Both the dynamic load profile and the HPPC profile recordthe cell’s reaction to high-power pulses, which may help us understand internal resistance and state-of-charge dynamics. The former model is more accurate to real-world driving situations with variable power demands. Improved ML model robustness and adaptability by training on these diverse profiles allows for precise SOC prediction in a broad variety of operational situations, including complicated and dynamic future scenarios.

**Fig. 5:**
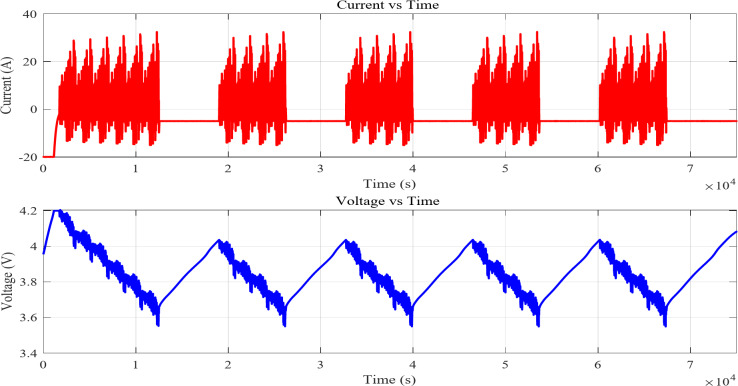
Current and voltage profile of dynamic profile.
  In renewable energy systems, electric cars, and grid storage, battery response to fluctuating power needs is crucial. The WLTP is a universally standardized test cycle created via international cooperation with the EU, Japan, India, Korea, and the USA. The analysis is based on empirical driving data amassed from 765,000 km spanning diverse geographies, road classifications, and circumstances. The cycle has three operational stages tailored to varying vehicle power-to-mass ratios. In conjunction with WLTP, it guarantees precise certification of emissions, fuel economy, and CO_2_ performance for light- duty cars^49^.
D. **High power pulse charging profile**
  **Fig. 6:**
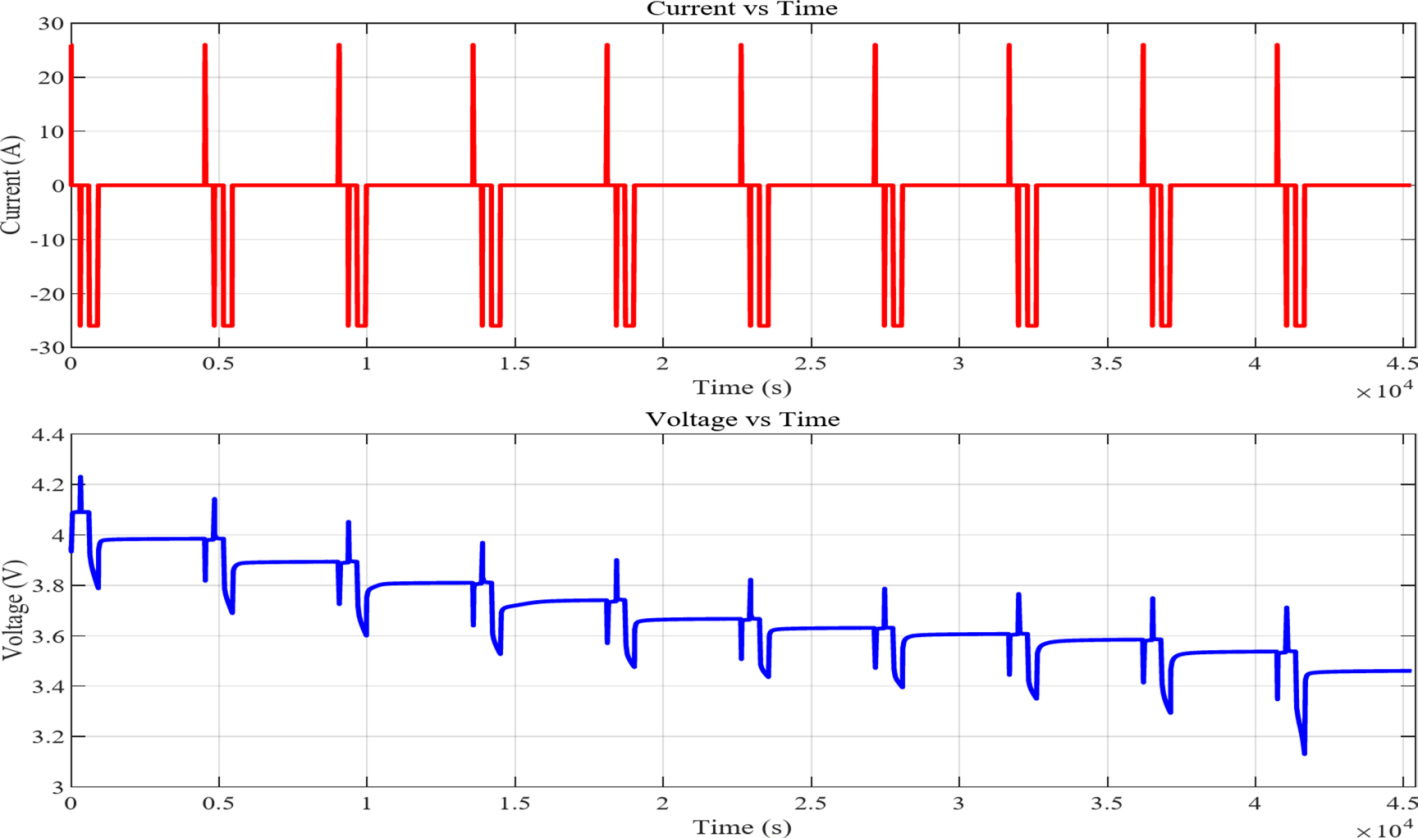
Current and voltage profile of HPPC.
  The HPPC profile uses high-power pulses to simulate sudden surges in high current demand or charging events. This profile tests the battery’s ability to charge or drain quickly without heat or performance loss. It helps evaluate battery performance in high-power applications like hybrid or electric automobiles, where quick charging or draining is common thing^50,51^. The current and voltage profile used in the load profile, as shown in Fig. 6. This HPPC test involves subjecting the cell to brief 10-second bursts of charging and discharging. The SOC is decreased by 10% after each pulse, and the cell is given 30 min to settle in between. Ten cycles of this procedure bring the battery’s state of charge down from 100 to 10%. The HPPC test is useful for evaluating the battery’s transient responsiveness, power capabilities, and internal resistance under pulse load circumstances. The test is composed of a series of discharge pulses (usually 10 s) at full discharge power, followed by a short rest time (180 s), then a charge pulse (10 s) at maximum regeneration power, and lastly a longer rest period (180 s or more) which is repeated at varying levels of state of charge^52^.
  SOC estimation uses classical and machine learning approaches. Coulomb Counting uses accumulated charge and discharge data to determine SOC, but cumulative errors over time reduce accuracy. The Extended Kalman Filter uses dynamic corrections based on a mathematical battery model to improve accuracy, but it requires precise system parameters. Linear Regression, SVM Regression, and Random Forest Regression estimate SOC using data. Linear Regression enhances older approaches but suffers with SOC prediction’s nonlinearity. SVM Regression models complicated correlations using kernel functions for improved accuracy but requires more processing resources. Random Forest Regression is the best method for SOC predictions because it uses ensemble learning to tackle nonlinearities, sensor noise, and outliers.
  The simulation findings show that although CC and EKF produce good estimates, machine learning approaches, notably Random Forest Regression, improve SOC forecast accuracy and resilience. The diversified dataset with distinct cycle and dynamic patterns assures that the trained models generalize effectively, making data-driven techniques attractive for exact SOC prediction in battery management systems.

A. **Full cycle profile**
  The battery charges and discharges six times. The battery is charged to maximum capacity and then drained to a predefined lower SOC. The battery’s ability to maintain efficiency, capacity, and performance is tested across six cycles. The machine learning model trained and evaluated using several driving cycles. These cycles generate various degrees of transients and nonlinearity in battery performance, enabling the model to generalize well across diverse settings. Additionally, its performance is further corroborated using a novel integrated dynamic loading profile, confirming its resilience in practical applications^47^. This profile shows the battery’s long-term performance and cycling- induced capacity loss. The following test procedure shows steps to the achieve the full cycling profile and is shown in Fig. 3.
  Constant Current and Constant Voltage discharges to 3 V and charges to 4.2 V at 1 C with a 10% of C rate cut-off current without rest intervals.
  This technique is repeated 6 times from a fully drained cell.
  This methodology uses 26 Ah Pouch cell to calculate cycle charge throughput.
  After these cycles, the CCCV procedure (1 C to 4.2 V, 10% of C rate cut-off current) completely charges the cell.
  **Fig. 3:**
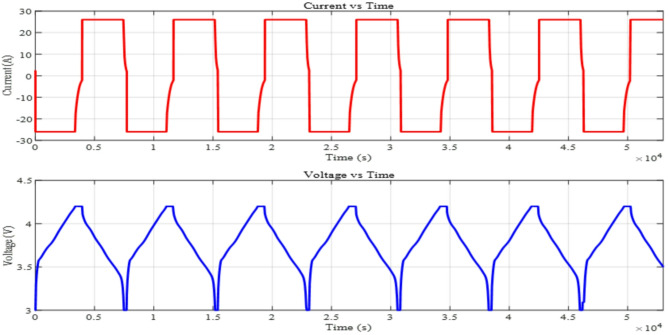
Current and voltage profile of full cycle.
B. **Partial discharge profile**
  In contrast to complete charge and discharge cycles, partial discharge profiles charge and drain the battery within a particular State of Charge range without reaching full capacity. In actual life, the battery may not always be fully charged or exhausted. It helps understand the battery’s performance during partial cycles, which are common in electric cars. While conventional methods often rely on offline analysis with extensive datasets or time-consuming full charge/discharge cycles, recent research has demonstrated a novel online estimation approach using partial discharge data.^48^.
  Figure 4 illustrates a battery cycling test, in which intermittent charging and discharging influence the voltage characteristics over time. The experimental cycling procedure included shallow cycling at a 1C rate without rest intervals, executed for a total of 24 cycles. The Partial cycles charge and discharge were conducted between 20% and 90% state of charge using a charge-controlled cycler operation.
  **Fig. 4:**
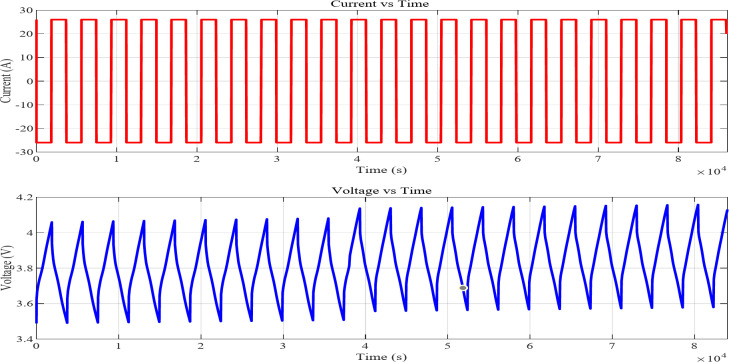
Current and voltage profile of partial cycle.
C. **Dynamic profile**.
  The dynamic profile simulates a battery with changing currents and power needs. Figure 5 shows the profile tests the battery’s performance in real time, including sudden load fluctuations, charging and draining events, and other dynamic factors.

## Results and discussion

**Fig. 7:**
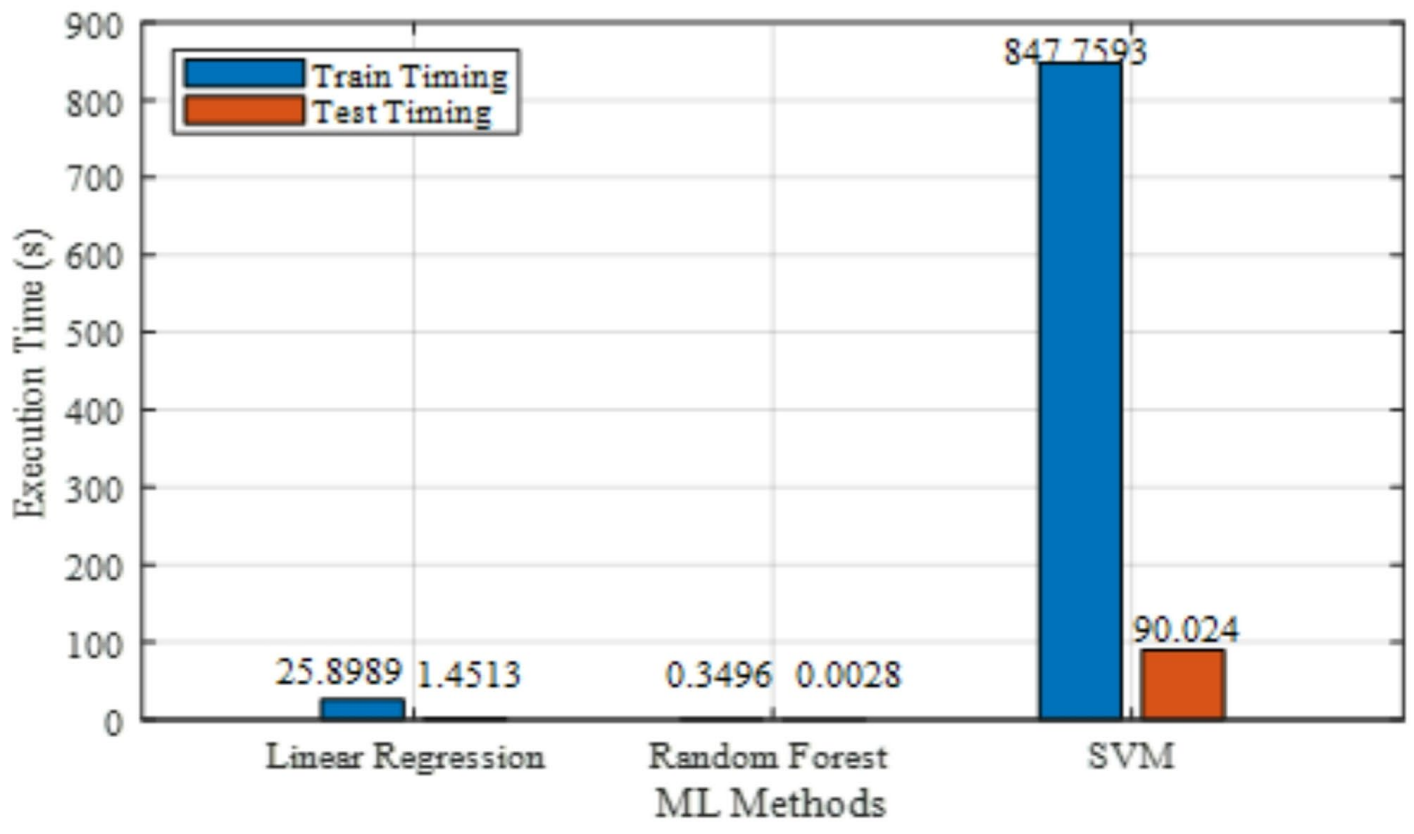
Execution time of machine learning methods.

The comparison of lithium-ion pouch cell state of charge estimate techniques by both traditional and machine learning method was analyzed. Figure 7 represents the execution time among the models; SVM is the most computationally costly due to its lengthy testing time of 90.02 s and greatest training time of 847.76 s. Finding a medium between speed and complexity, Linear Regression takes 25.90 s to train and 1.45 s to test. By comparison, Random Forest has the quickest execution speeds out of the three. Training takes only 0.35 s and testing takes virtually nothing at 0.0028 s. According to these findings, Support Vector Machines shine in situations where precision is more important than speed, Random Forest shines in situations when quick computation is required, and Linear Regression strikes a good compromise between training efficiency and model complexity.

**Fig. 8:**
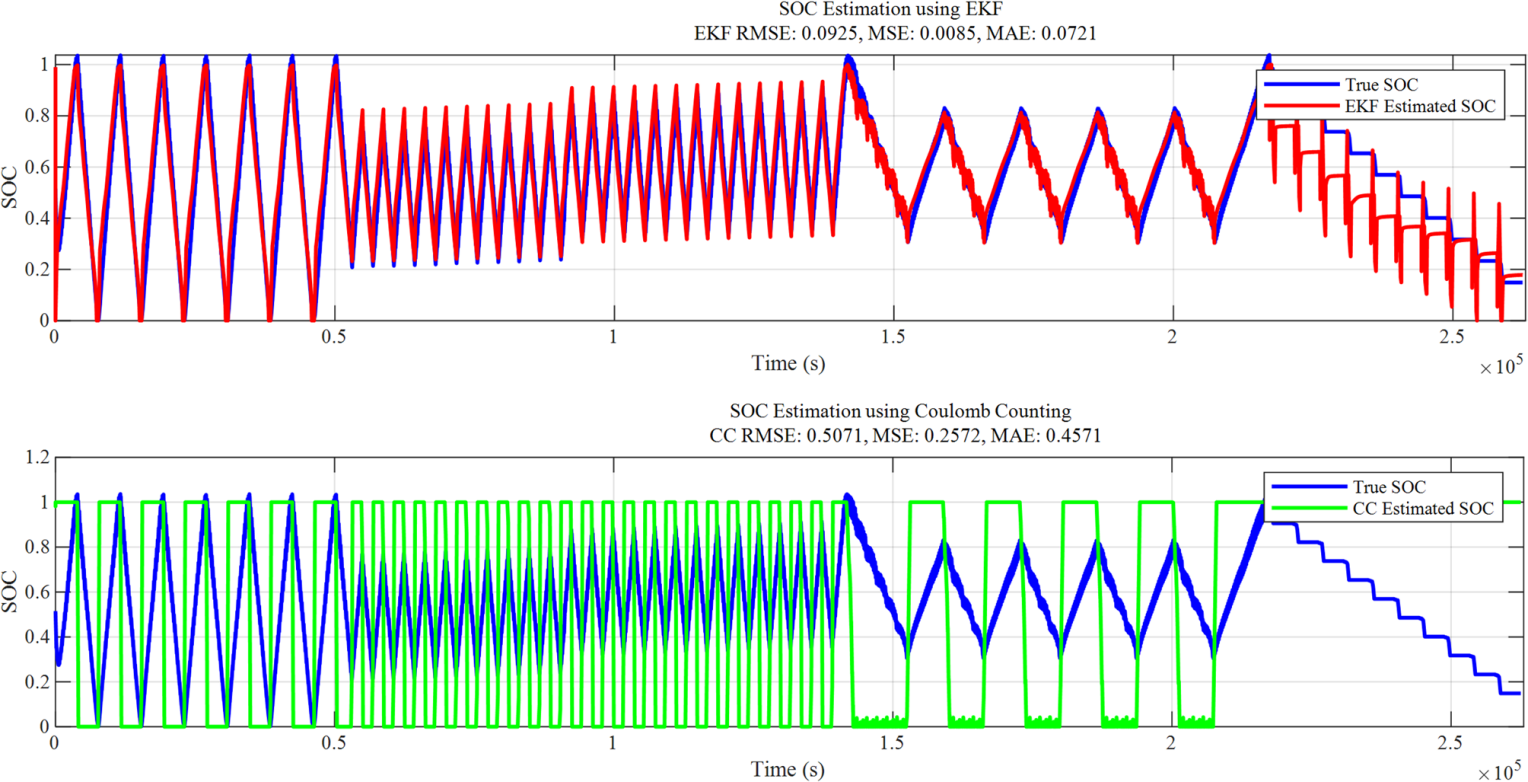
SOC estimated by Coulomb counting and EKF method.

Table 2 provides a comparative examination of several approaches for State of Charge estimation, using RMSE, MSE, and MAE as metrics. Figure 8 demonstrate that Coulomb Counting exhibits the highest errors (RMSE: 0.5071, MSE: 0.2572, MAE: 0.4571), highlighting its limitations due to cumulative errors over time caused by sensor noise and inaccuracy. The Extended Kalman Filter improves accuracy (RMSE: 0.0925) by using a model-based estimation for dynamic correction; however, it requires detailed system modeling. Linear Regression (RMSE: 0.0778) demonstrates further enhancement but struggles due to the nonlinear characteristics of SOC. SVM Regression (RMSE: 0.0319) achieves lower errors by modeling intricate correlations using kernel functions, though it incurs greater computational costs.

**Table 2:** Error metrics for different methods.

Method	RMSE	MSE	MAE
Coulomb counting	0.5071	0.2572	0.4571
EKF	0.0925	0.0085	0.0721
Linear regression	0.0778	0.0061	0.0518
SVM regression	0.0319	0.0010	0.0194
**Random Forest**	**0.0229**	**0.0005**	**0.0139**

**Fig. 9:**
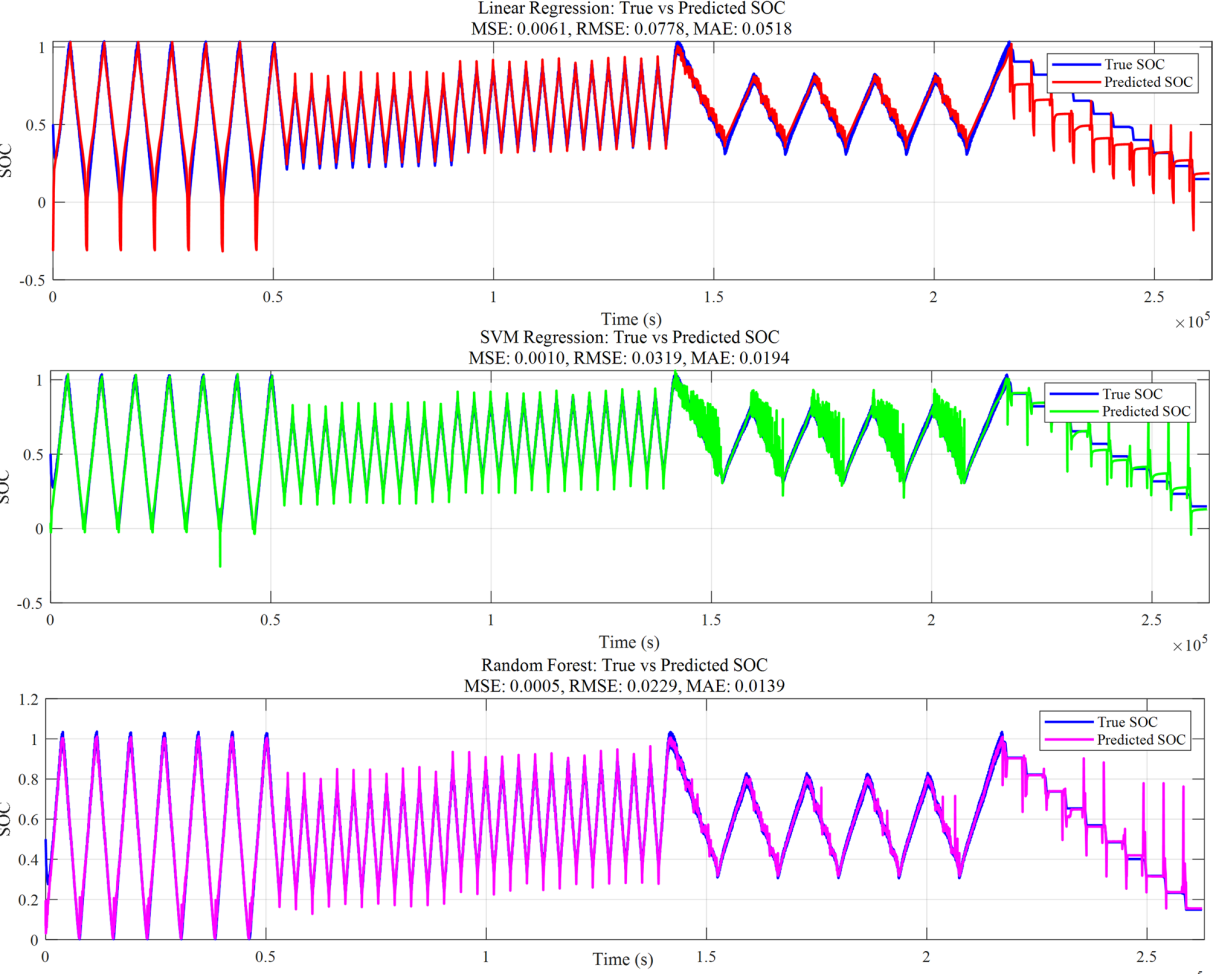
SOC estimated by machine learning methods.

The most effective approach is Random Forest Regression, achieving the lowest errors (RMSE: 0.0229, MSE: 0.0005, MAE: 0.0139). Its ensemble learning technique enables it to efficiently handle nonlinearity, noise, and outliers, making it the most suitable choice for SOC estimation. Overall, machine learning techniques, particularly Random Forest, outperform traditional methods, making them more appropriate for accurate SOC estimation. The comparison of lithium-ion pouch cell state of charge estimate techniques is show in Figs. 8 and 9 indicates significant trade- offs between old and new methodologies. The EKF successfully combines system dynamics and feedback correction, performing well under varying loads despite some oscillations during transitions; whereas Coulomb Counting’s simple current integration approach suffers from cumulative errors that limit its long-term reliability.

Machine learning approaches enhance accuracy, with Linear Regression giving a fundamental answer, Support Vector Machine Regression better managing nonlinear behavior, and Random Forest reaching the maximum accuracy via ensemble learning. However, although these ML techniques are more accurate, they need significant computing resources and training data, posing obstacles for real-time deployment.

## Conclusion

The research definitively shows that machine learning-based State of Charge prediction, especially using Random Forest Regression, offers substantial benefits compared to conventional techniques like Coulomb Counting and the Extended Kalman Filter. The Random Forest model utilizes ensemble learning to accurately represent the non-linear characteristics of lithium-ion batteries while substantially reducing the impact of sensor noise and parameter fluctuations. This yields much better accuracy, as shown by the lowest error metrics (RMSE = 0.0229, MSE = 0.0005, MAE = 0.0139), and increased flexibility to fluctuating operating circumstances. Unlike conventional approaches that are hindered by accumulated mistakes or need exact battery specifications, the machine learning methodology derives insights directly from the data, making it more robust against aging effects and environmental fluctuations. Thus, the ML-based SOC estimate is exceptionally appropriate for practical battery management systems in electric cars and energy storage applications, ensuring enhanced system efficiency and extended battery longevity.

Advanced machine learning approaches like deep learning and ensemble learning methods might improve SOC prediction in the future. Additionally, incorporating different types of load profiles can further enhance the robustness of SOC estimation. These diverse load conditions allow models to generalize better and improve prediction accuracy under varying operating scenarios.

## Data Availability

Data will be available on request from the corresponding author.
